# To forage, mate, or thermoregulate: Influence of resource manipulation on male rattlesnake behavior

**DOI:** 10.1002/ece3.3193

**Published:** 2017-07-20

**Authors:** Sasha J. Tetzlaff, Evin T. Carter, Brett A. DeGregorio, Michael J. Ravesi, Bruce A. Kingsbury

**Affiliations:** ^1^ Department of Natural Resources and Environmental Sciences University of Illinois at Urbana‐Champaign Urbana IL USA; ^2^ Department of Ecology and Evolutionary Biology University of Tennessee Knoxville TN USA; ^3^ US Army ERDC‐CERL Champaign IL USA; ^4^ Michigan Department of Military and Veterans Affairs Grayling MI USA; ^5^ Department of Biology Indiana‐Purdue University Fort Wayne IN USA

**Keywords:** body condition, food supplementation, mate searching, resource selection, *Sistrurus catenatus*, thermoregulation

## Abstract

Male animals should preferentially allocate their time to performing activities that promote enhancing reproductive opportunity, but the need to acquire resources for growth and survival may compete with those behaviors in the short term. Thus, behaviors which require differing movement patterns such as ambushing prey and actively searching for mates can be mutually exclusive. Consequently, males that succeed at foraging could invest greater time and energy into mate searching. We radio‐tracked sixteen male massasauga rattlesnakes (*Sistrurus catenatus*) and supplemented the diets of half the snakes with mice across an active season. We tested the predictions that reduced foraging needs would allow fed snakes to move (i.e., mate search) more, but that they would consequently be stationary to thermoregulate less, than unfed controls. Contrary to our first prediction, we found no evidence that fed snakes altered their mate searching behavior compared to controls. However, we found controls maintained higher body temperatures than fed snakes during the breeding season, perhaps because fed snakes spent less time in exposed ambush sites. Fed snakes had higher body condition scores than controls when the breeding season ended. Our results suggest the potential costs incurred by devoting time to stationary foraging may be outweighed by the drive to increase mating opportunities. Such instances may be especially valuable for massasaugas and other temperate reptiles that can remain inactive for upwards of half their lives or longer in some cases, and for female rattlesnakes that generally exhibit biennial or more protracted reproductive cycles.

## 1 INTRODUCTION

Male animals should allocate time to activities that will help them optimize reproductive opportunity (Parker, 1974). However, the drive to acquire resources for growth and survival may compete with those behaviors in the short term. This conflict is observed in a variety of animal species wherein free‐ranging males cannot forage and simultaneously pursue mating opportunities or guard mates. For instance, male baboons (*Papio cynocephalus*) moved and foraged less while mate guarding (Alberts, Altmann, & Wilson, 1996). Provisioning animals with food can affect their foraging and reproductive behavior. Food‐supplemented male red‐winged blackbirds (*Agelaius phoeniceus*) spent less time foraging, more time guarding females, and sired more offspring (Westneat, 1994). Similar conflicts related to energy acquisition and mating have been observed for ectotherms. Male guppies (*Poecilia reticulata*) studied under laboratory conditions first foraged to ensure they had sufficient energy reserves before courting females (Abrahams, 1993). Many terrestrial ectotherms likely face the additional challenge of balancing the need to forage and mate with the need to behaviorally thermoregulate. Here we conducted food supplementation experiments with males of a temperate pit viper species to better understand what trade‐offs may exist between these behaviors.

Male ectotherms may sacrifice acquiring certain resources if the costs outweigh the benefits or if their needs cannot be met due to time or energy budget constraints. Most viperid snakes hunt predominantly by ambush (i.e., sit‐and‐wait foraging). This largely stationary foraging tactic may reduce the available time to move to find mates, as some male vipers feed during the breeding season (Tetzlaff, Ravesi, Parker, Forzley, & Kingsbury, 2015) or may even increase feeding frequency compared to the nonbreeding season (Webber, Glaudas, & Rodrıguez‐Robles, 2012). As such, males of numerous snake species decrease feeding rates (Glaudas & Alexander, 2017) or fast (Daltry, Wuster, & Thorpe, 1998; Slip & Shine, 1988) during the breeding season, suggesting foraging may come at the expense of increasing reproductive opportunities for these species. Male snakes which have built up energy reserves from feeding could invest substantial effort into finding females, given the high energetic investment of reproduction (Bonnet & Naulleau, 1996; Olsson, Madsen, & Shine, 1997).

Behavioral thermoregulation is important for regulating nearly all physiological processes for ectotherms (Angilletta, Niewiarowski, & Navas, 2002), but like foraging trade‐offs, snakes may sacrifice thermoregulating to increase mating prospects. For example, male red‐sided gartersnakes (*Thamnophis sirtalis parietalis*) forgo thermoregulatory opportunities and instead focus on courting females during the mating season (Shine, Harlow, Elphick, Olsson, & Mason, 2000). Thermoregulation appears to be more compatible for snakes that hunt by ambush as opposed to those that actively search for prey, because the former can select ambush sites that also facilitate thermoregulation (Harvey & Weatherhead, 2011).

As both stationary foraging and thermoregulation likely conflict with mate searching, these behaviors have fitness costs and benefits for snakes (Shine, 2003). We evaluated the hypothesis that stationary foraging and thermoregulatory needs constrain male ambush‐hunting snakes’ ability to mate search. We accomplished this by supplementing the diets of male massasauga rattlesnakes (*Sistrurus catenatus*) with mice across an active season. Manipulating food resources has been a useful technique for observing behavioral responses of foraging, thermoregulation, movement, and reproduction for numerous terrestrial vertebrates (Bacigalupe, Rezende, Kenagy, & Bozinovic, 2003; Boutin, 1990), including snakes (Blouin‐Demers & Weatherhead, 2001; Glaudas & Alexander, 2017; Taylor, Malawy, Browning, Lemar, & DeNardo, 2005; Wasko & Sasa, 2012). As food‐supplemented males should have minimal or no need to allocate time to stationary foraging, we tested the prediction that these snakes would move more during the breeding season than males which were not food supplemented. However, we predicted this would come at the expense of stationary thermoregulation and that supplemented snakes would consequently maintain lower body temperatures than unfed snakes. We also assumed that supplemented snakes would consume more food and would therefore be in better body condition than unfed snakes after the breeding season ended.

## 2 METHODS

### 2.1 Study species and site

**Figure 1:**
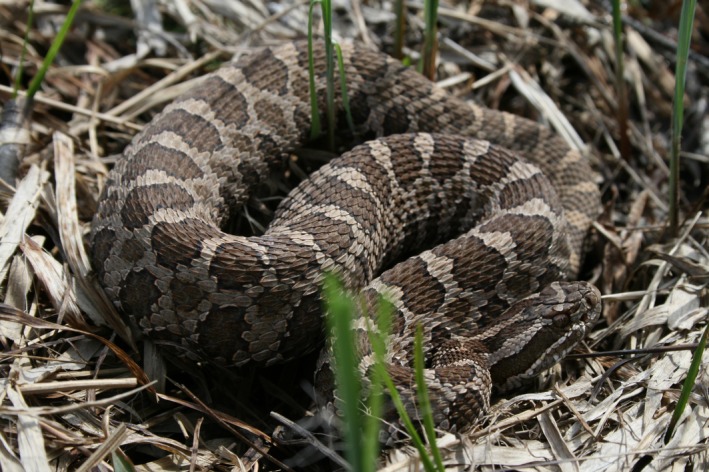
The massasauga (*Sistrurus catenatus*) is a temperate rattlesnake that feeds primarily on small mammals Photograph by Sasha J. Tetzlaff

The massasauga is an ambush‐hunting rattlesnake (Figure 1) native to much of the Great Lakes region of the United States and southern Canada. The species was recently listed as threatened under the Endangered Species Act of the United States of America due to habitat loss, road mortality, and persecution (U.S. Fish and Wildlife Service 2016). These snakes have a prolonged polygynous mate searching system, where males cover large areas in search of numerous widely dispersed females during the ca. 1‐ to 2‐month breeding season, which generally occurs during mid to late‐summer (DeGregorio, Manning, Bieser, & Kingsbury, 2011; Jellen, Shepard, Dreslik, & Phillips, 2007; Tetzlaff, Ravesi, & Kingsbury, 2016). We studied male massasaugas from a population approaching the northern range limit for the species (44°38′9.19″N, 84°53′21.94″W) from May to August 2014 in Michigan, USA at Camp Grayling Joint Maneuver Training Center, which is the largest National Guard training facility in the United States. Our specific study site was a ca. 800 ha portion of Camp Grayling less disturbed by military operations. The site consists primarily of patchy forests, including coniferous and hardwood communities comprised of spruce *(Picea* spp.), cedar (*Thuja* spp.), pine (*Pinus* spp.), maple (*Acer* spp.), oak (*Quercus* spp.), and aspen (*Populus* spp.) stands. There are also extensive patches of scrub‐shrub wetlands comprised of willow (*Salix* spp.) and speckled alder (*Alnus incana*), as well as smaller open areas (referred to as barrens) that are dominated by lichen and blueberry (*Vaccinium* spp.). Finally, there are several areas that were traditionally forested but are open‐canopied due to anthropogenic disturbance such as logging and burning.

### 2.2 Radio‐telemetry and feeding experiments

We captured adult male massasaugas from May to June 2014 and surgically implanted a temperature sensitive radio‐transmitter (model SB‐2T or SI‐2T, Holohil Systems, Ltd., Ontario, Canada) into the body cavity of snakes using adapted methods of Reinert and Cundall (1982). We recorded the mass (g) and snout‐to‐vent length (SVL; cm) for each snake when it was anesthetized at the time of transmitter implantation. A total of sixteen males were included in the study. Eleven were outfitted with transmitters in 2014, eight of which were food supplemented (hereafter termed “fed snakes”). The other three males incorporated into the study in 2014 as well as an additional five that were already being radio‐tracked since 2013 as part of a concurrent, larger radio‐telemetry study (Ravesi, 2016) served as naturally foraging controls and were not offered supplemental food. We randomly assigned each snake incorporated into the study in 2014 to a fed or control treatment. All controls were implanted with a 9‐g transmitter. All fed snakes were implanted with a 5‐g transmitter and a 3‐g temperature logger (Thermochron iButton, model DS1921G, Fondriest Environmental, Inc.) for a concurrent study investigating the postprandial response of snakes to feeding (Tetzlaff, 2015). Each temperature logger was programmed to record body temperature (*T*_b_) every 1–2 hr, dependent on when the logger was implanted as they are limited in the number of data points they can store. Point versus semi‐continuous *T*_b_ sampling can affect inferences (Taylor, DeNardo, & Malawy, 2004). We therefore extracted *T*_b_s obtained from semi‐continuous loggers for fed snakes to match them by time to the *T*_b_s obtained from transmitter point samples for controls in our thermoregulation analysis (see below). Although only fed snakes were implanted with a temperature logger, the total mass of implanted transmitters and/or loggers never exceeded 6% of body mass for a given individual.

We defined the breeding season as 14 July–18 August according to the first and last observed behaviors assumed to be related to reproduction, such as male–female pairings and markedly increased movement rates by males (DeGregorio et al., 2011; Harvey & Weatherhead, 2006; Jellen et al., 2007). We periodically located snakes prior to the mating season using a handheld receiver (R‐1000, Communications Specialists, Inc., Orange, CA, USA) and three element Yagi antenna to keep track of their spatial distributions and to conduct feeding experiments. We located each individual every 48 hr on average during the mating season, and we varied the time of day each individual was tracked for subsequent radio‐telemetry events. We recorded the position of each snake when located in Universal Transverse Mercator units (North American Datum of 1983) with a handheld GPS (Garmin eTrex 30; 3 m accuracy).

We offered each fed snake two thawed, commercially raised mice (ca. 20 g each) from a pair of snake tongs *in situ* every 10–14 days from 21 May to 14 August 2014. We offered supplemental food to snakes prior to the mating season so they had the opportunity to build up energy reserves before it began. We initiated supplemental feeding for snakes as soon as they were assigned to the treatment group. Because snakes were implanted with transmitters opportunistically as we captured them, the timing of their first food supplementation event ranged from 12 to 54 days prior to the breeding season. Throughout the active season, all snakes were fed on the same days. Despite the staggered initiation of first food supplementation, we restricted all analyses to behavior occurring after the initiation of the breeding season (14 July). When a snake was presented with a food item and showed interest in feeding, it was observed from a distance until one or both mice were eaten (generally within an hour) so we could be certain how much food was consumed per feeding event. We fed snakes only during daylight hours (0900–2000).

### 2.3 Breeding season movement

We calculated movement rate (m/day) for each snake during the mating season as the cumulative distance moved (Euclidean distances between successive radio‐telemetry locations) divided by the number of days monitored. We used a linear mixed model (LMM) to compare movement rates between fed and control snakes. We included snake ID as a random effect to control for individual variation in movement. We also included the cumulative amount of supplemental food consumed by fed snakes up to the point of a given telemetry event as a random effect due to variation in the number of successful supplemental feeding events per snake.

We created 95% minimum convex polygons (MCPs) around the outermost radio‐telemetry locations for each snake in ArcGIS 10.1 to evaluate the total area (ha) traversed by snakes throughout the breeding season. These MCPs were not used to determine an individual's home range but rather were used to quantify the amount of area they traversed during the breeding season as a complement to movement rate. Creating MCPs for each snake should accurately reflect their space use during the period of interest because all snakes were tracked at the same frequency throughout the breeding season. We used the same general LMM approach outlined above to test whether fed and control snakes differed in the size of area traversed within the study site.

### 2.4 Thermoregulation

We obtained *T*_b_s of fed snakes from their internal temperature loggers as noted above. We collected two sets of pulse rates from each control snake's temperature sensitive radio‐transmitter during each telemetry event and averaged them. These mean pulse rates were transformed to *T*_b_s using third‐ or fourth‐order polynomial regression equations from calibration curves supplied by the transmitter manufacturer (*R* ^2^ ≥ .99 for all equations).

We placed 10 physical models of massasaugas throughout the study site to collect hourly operative environmental temperatures (*T*_e_) that approximate *T*_b_s of snakes in the absence of clear behavioral or physiological thermoregulation (i.e., thermoconformity) (Blouin‐Demers & Weatherhead, 2001; Carter, Eads, Ravesi, & Kingsbury, 2015; Harvey & Weatherhead, 2011). The models were 10‐cm‐long and 2.54‐cm‐diameter copper tubes which we painted dark brown to mimic the thermal properties of a massasauga. We suspended the same type of temperature logger implanted in fed snakes inside each tube, filled them with water, and sealed them with rubber stoppers and silicone. See Tetzlaff (2015) for further details of model construction and validation. We moved each model to a new area within the study site every 10 days to capture variation in the thermal environment that the snakes could have experienced.

We used a LMM fit by maximum likelihood to test whether fed snake and control snake *T*_b_s differed. We included the *T*_e_ at the time a snake was located as a covariate to control for the variation in the environmental temperatures that can influence *T*_b_s. We included snake ID as a random effect because repeated *T*_b_s collected for a given individual are not independent. We also included the cumulative amount of supplemental food consumed up to a given telemetry event as a random effect because accumulated fat reserves from food supplementation could conceivably affect thermoregulatory behavior. We included *T*_b_s and *T*_e_s collected only during 0900–2000 in our analysis as snakes were located and fed during daylight hours.

### 2.5 Body condition

We recorded an initial mass and SVL for all males when they were anesthetized for transmitter implantation (before food supplementation began) and a final mass and SVL for those that we could recapture after the breeding season ended. We recorded final mass and SVL on anesthetized fed snakes when their transmitters were removed in the laboratory, but we recorded these data *in situ* for conscious controls as they were still being radio‐tracked for a concurrent study (Ravesi, 2016). Thus, we used the mean of each snake's initial and final SVL when calculating body condition indices to better account for the potential discrepancies between the precision of measurement techniques.

We created an initial body condition index (BCI) for each snake from the residuals of a general linear model using each snake's log‐transformed initial mass and log‐transformed mean SVL. We calculated each snake's final BCI as the difference of the log‐transformed final mass and its predicted initial BCI score. We used two‐sample *t* tests to determine whether initial as well as final BCI differed between treatments. As snakes in each treatment were radio‐tracked for differing lengths of time, we used a general linear model to determine whether the interaction of treatment group and number of months radio‐tracked predicted final BCI. We also used general linear models to examine whether the amount of supplemental food consumed by fed snakes predicted change in their BCI scores as well as their final BCI.

### 2.6 General procedures

All procedures were conducted under an animal care and use protocol approved by Purdue University (#1112000451) and a Michigan Department of Natural Resources Scientific Collector's Permit. We conducted all statistical tests using R version 3.1 (R Core Team 2014) with α set at 0.05. We used the lme4 package (Bates, Maechler, Bolker, & Walker, 2015) in R for analyses using mixed models. Parameter estimates and means are reported ± standard error (*SE*) unless otherwise noted.

## 3 RESULTS

### 3.1 Radio‐telemetry and feeding experiments

We radio‐tracked eight fed snakes 116 times and eight controls 97 times during the breeding season. The mean mass ± standard deviation (*SD*) and SVL ± *SD* at the time of transmitter implantation was 206 ± 38 g and 57 ± 5 cm for fed snakes and 191 ± 47 g and 56 ± 6 cm for controls. There was no potential size advantage for snakes in either treatment that may have affected any of the behavioral metrics we examined as fed snakes did not differ from controls in initial mean mass (*t* = −0.722, *p = *.482) or SVL (*t* = −0.452, *p *=* *.658). Overall, fed snakes consumed a mean ± *SD* of 140.3 ± 77.4 g of food (7 ± 3.9 mice) in 37 total feeding events from 21 May to 14 August 2014, with a range of 20 g (1 mouse) to 280 g (14 mice) consumed per individual (Table 1). The minimum and maximum meal size consumed was 7.5% and 24% of a given snake's mass, respectively.

**Table 1:** Snake identification, number of times each snake was food‐supplemented, and total amount of supplemental food consumed for *Sistrurus catenatus* during 2014 in Grayling, Michigan, USA

ID	Feeding events	Food consumed (g)
F1	8	280
F2	6	182
F3	4	100
F4	5	140
F5	1	20
F6	5	160
F7	5	160
F8	3	80

### 3.2 Breeding season movement

There was a great deal of individual variation in movement indices for both fed and control snakes during the breeding season. Daily movement rates of fed snakes ($\overset{¯}{x}$ = 74.6 ± 13.7 m/day) did not differ significantly from controls ($\overset{¯}{x}$ = 97.2 ± 18.7 m/day; β = −22.6 ± 73.6, *z* = −0.308, *p *=* *.986). These movement rates were similar to breeding season movements previously reported for massasaugas at Camp Grayling (48–72 m/day: DeGregorio et al., 2011). The area traversed by fed snakes ($\overset{¯}{x}$ = 7.5 ± 1.8 ha) also did not differ significantly from controls ($\overset{¯}{x}$ = 9.0 ± 3.4 ha; β = −1.5 ± 7.2, *z* = −0.212, *p *=* *.996).

### 3.3 Thermoregulation

**Figure 2:**
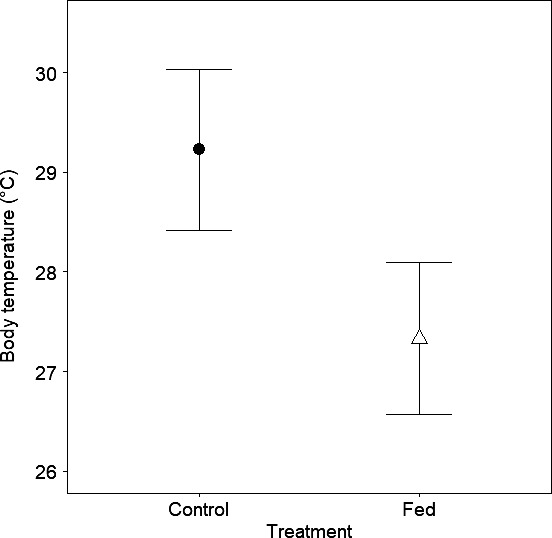
Predicted mean body temperatures modeled using operative environmental temperature as a covariate with 95% confidence limits for naturally foraging (control; filled circle) and food‐supplemented (fed; open triangle) *Sistrurus catenatus* during the 2014 breeding season in Grayling, Michigan, USA

When accounting for *T*_e_ at the time each *T*_b_ was recorded, the diurnal *T*_b_s of fed snakes differed significantly from those of controls (β* = *−1.894 ± 0.564, *t = *−3.358, *p = *.001). Fed snake predicted *T*_b_s ($\overset{¯}{x}$ = 27.33, 95% confidence interval: 26.57–28.09) were lower than controls ($\overset{¯}{x}$ = 29.22, 95% confidence interval: 28.41–30.04; Figure 2).

### 3.4 Body condition

**Figure 3:**
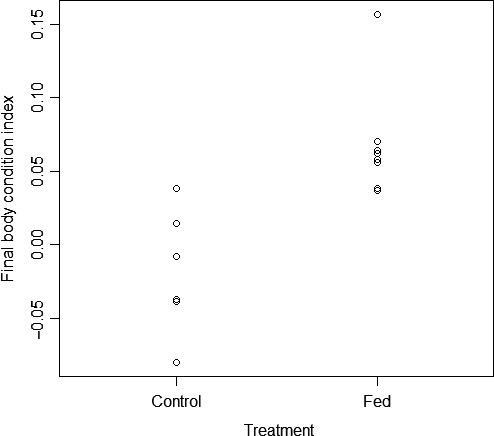
Final body condition scores based on the residuals of mass and snout‐to‐vent length for naturally foraging (control) and food‐supplemented (fed) *Sistrurus catenatus* at the conclusion of the 2014 breeding season in Grayling, Michigan, USA

**Figure 4:**
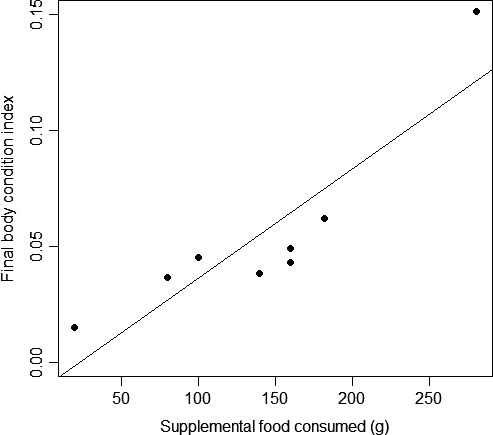
Final body condition scores based on the residuals of mass and snout‐to‐vent length for food‐supplemented *Sistrurus catenatus* at Grayling, Michigan, USA as a function of the total amount of supplemental food consumed per snake.

We recorded a final mass and SVL for all fed snakes after the breeding season, but we recorded these morphometric data for only six controls; one was killed by a vehicle while crossing a paved road, and the transmitter failed on another in August 2014. Initial BCI scores of fed snakes did not differ significantly from controls before food supplementation began (*t = *−0.935, *p = *.379), but fed snakes had significantly higher final BCI scores than controls (*t = *−3.943, *p = *.003; Figure 3). The interaction of treatment and number of months each snake was radio‐tracked was not a significant predictor of final BCI (β* = *0.049 ± 0.025, *t = *1.946, *p = *.080). The amount of supplemental food consumed by fed snakes was a significant positive predictor of change in BCI scores (*R* ^2^ *= *.590, *F*_1,6_ = 8.593, *p = *.026) as well as final BCI (*R* ^2^ *= *.789, *F*_1,6_ = 22.46, *p = *.003; Figure 4).

## 4 DISCUSSION

We predicted that by providing snakes with food which came at no additional time or energy cost to acquire, they would compensate by spending less time foraging and more time actively mate searching. Contrary to our prediction, we found no evidence that fed snakes moved greater daily distances or traversed larger areas than controls. Movement is critical to mate acquisition for male massasaugas, and males that move more encounter more females (Jellen et al., 2007). We assumed all the males we radio‐tracked were engaging in mate searching behavior, but this may not have been true. Although male snakes typically reproduce annually, this is not always the case (Shine, 2003). We observed instances of males in both treatments in proximity to, courting, and copulating with females. However, due to the nature of radio‐telemetry studies, we do not know how many individuals acquired females or successfully mated. Massasaugas have been studied at Camp Grayling for over a decade, and the population appears to be dense and robust (BA Kingsbury et al., personal observation). Males searching for females at this site may not have to travel far to find a female. Although we did not notice fed snakes moving more than controls, this may be a function of the distribution of females and not an accurate reflection of their mate searching behavior.

Snakes have lower metabolic rates and feed less often than comparably sized endotherms (Beaupre & Duvall, 1998; Nagy, 2005). Foraging pressures therefore may not have been particularly strong on controls, leading to our inability to detect differences in movement. Alternatively, because fed snakes continued to consume supplemental food during the breeding season, stationary digestion may have ultimately resulted in movements comparable to controls. Furthermore, the benefits of increased movements for all snakes may have been outweighed by its potential costs given that movement can intensify exposure to predators, limits stationary thermoregulatory opportunities, and may lead to unnecessary energy expenditure (reviewed in Shine, 2003).

The lack of differences in movement we observed between fed and control snakes may not be indicative of a lack of difference but rather simply in our ability to discern a difference in behavior for this particular species. Despite being primarily an ambush hunter, massasaugas appear to remain at ambush sites for short periods of time, as we rarely observed snakes in the same location during subsequent radio‐tracking events (SJ Tetzlaff and MJ Ravesi, personal observation). Discerning differences in movements for snakes assumed to primarily be engaged in mate searching (fed snakes) and those that may be frequently moving between ambush sites (controls) could be difficult. In a detailed study of foraging behavior of northern pacific rattlesnakes (*Crotalus oreganus*), Putman, Barbour, and Clark (2016) noted the majority of snakes spent <5 hr at ambush sites. This “impatient ambushing” foraging tactic may coincide with mate searching for snakes that continue to feed during mating periods, which has been observed for both sexes of massasaugas at Camp Grayling (Tetzlaff et al., 2015). Other vipers that commonly remain at an ambush site for up to several days (e.g., bushmaster, *Lachesis muta*; Greene, 1986) may be better candidates for food supplementation studies investigating the spatial ecology of supplemented and naturally foraging snakes.

Researchers comparing general movement patterns of food supplemented and naturally foraging vipers have reported contrasting results. Taylor et al. (2005) found supplementing the diets of female western diamond‐backed rattlesnakes (*Crotalus atrox*) in Arizona, USA yielded no difference in home range size compared to naturally foraging conspecifics. Wasko and Sasa (2012) determined both sexes of terciopelos (*Bothrops asper*) that were food supplemented in a prey‐limited environment in Costa Rica did not differ in home range sizes, but supplemented snakes had shorter and less frequent movements than unfed controls. Glaudas and Alexander (2017) found naturally foraging male puff adders (*Bitis arietans*) also did not differ in home range size but moved farther within defined home ranges to find ambush sites compared to food‐supplemented males. These results suggest that food supplementation may affect the movements of vipers in ways that vary geographically, temporally, and demographically. However, a consistent pattern across these studies, including ours, is that food supplementation leads either to higher body condition, increased growth rates, or greater mass gain compared to unfed snakes.

Our prediction that fed snakes would maintain lower *T*_b_s than controls was supported. We expected this would be explained by the assumption that controls would move less and invest more time to stationary foraging than fed snakes—a behavior that is compatible with thermoregulation for ambush‐hunting massasaugas (Harvey & Weatherhead, 2011). This appeared to not be the case based on our movement results, and the behavior of snakes in each treatment may have differed when they were not moving. The benefits to thermoregulation may be outweighed by its costs under certain contexts. Massasaugas have been observed to be at most only partially concealed when hunting (Harvey & Weatherhead, 2011, SJ Tetzlaff, personal observation). Thus, there may have been a thermoregulatory trade‐off for the presumably satiated fed snakes in our study with respect to behaviors which we did not study, such as predator avoidance, if fed snakes were exposed less often than controls.

Although controls might have spent more time in exposed ambush sites (because they maintained higher *T*_b_s) than fed snakes, they appeared to not consume as much food overall as they had lower body condition scores than fed snakes after the breeding season. We fed and radio‐tracked males for only one active season, but the additional benefits of food supplementation beyond the immediate ones (e.g., reducing the need to ambush hunt and increasing energy reserves) may be more apparent in subsequent active seasons, given that numerous ectothermic taxa rely on energy reserves based on “capital” for reproduction (Bonnet, Bradshaw, & Shine, 1998). This viewpoint corroborates our body condition results, as it could be expected that fed snakes would have used much of the energy gained from supplemental food if they had moved more than controls, thus potentially negating the differences we detected in body condition between treatments. Condition‐dependent reproductive investment is believed to be a trade‐off between current and future reproductive success. For example, Lind and Beaupre (2015) studied male timber rattlesnakes (*Crotalus horridus*) across four active seasons and found individuals with higher energy stores invested greater effort into mate searching, whereas those with lower body condition spent more time foraging during the breeding season. Continuing studies such as ours over multiple active seasons should reveal more apparent effects on life history, as was documented for food‐supplemented female rattlesnakes that had an overall much higher incidence of reproduction than unfed females (Taylor et al., 2005).

Our sample sizes were small and potentially limited our ability to discern differences in behavior between fed and control snakes. However, the lack of observed differences in movement patterns is intriguing and provides insight into the behavior of massasaugas, which appears to not be constrained by energy acquisition, at least in the short term. We assumed that fed snakes would immediately shift their time from foraging to mate searching when the need to forage was reduced due to the short active season (generally 6 months) of this high latitude species. We further predicted that the movement patterns of foraging and mate searching snakes would be clearly different because massasaugas are ambush hunters. However, this prediction was unsupported due to either an inability to discern the difference between the movement patterns of foraging snakes and mate searching snakes or the absence of a true difference between behavior of fed and unfed snakes. Female rattlesnakes are generally not receptive to mating annually and often exhibit biennial (e.g., *C. atrox*; Tinkle, 1962), triennial, or even quadrennial reproductive cycles (e.g., three–four years on average for *C. horridus*; Brown, 1991, 2016). This includes massasaugas at our study site (SJ Tetzlaff, personal observation). Male rattlesnakes need to forage, thermoregulate, avoid predators, and find receptive females to mate with during a given active season. Our results suggest that males at this site can balance satisfying several resource needs and that reducing the need to forage results in relatively little change in behavior. The question of whether we should even expect differences in behavior of food‐supplemented snakes and naturally foraging conspecifics will be easier to answer with detailed (and ideally simultaneous) documentation of natural feeding rates (e.g., Glaudas & Alexander, 2017; Putman et al., 2016), annual energy budgets, and the energetic demands associated with the behaviors we studied (Lind & Beaupre, 2015). More research with a diverse range of ectothermic taxa is needed to further understand how these animals prioritize energy acquisition and resource use, especially considering the ever‐increasing threats to their persistence such as habitat loss, disease, and climate change.

## CONFLICT OF INTEREST

None declared.
